# Thirty-five years (1986–2021) of HIV/AIDS in Nigeria: bibliometric and scoping analysis

**DOI:** 10.1186/s12981-022-00489-6

**Published:** 2022-12-21

**Authors:** Henshaw Uchechi Okoroiwu, Ekementeabasi Aniebo Umoh, Edet Effiong Asanga, Uwem Okon Edet, Michael Raymond Atim-Ebim, Edum Abang Tangban, Elizabeth Nkagafel Mbim, Cynthia Amarachi Odoemena, Victor Kanu Uno, Joseph Okon Asuquo, Otu Otu Effiom-Ekaha, Ogechukwu C. Dozie-Nwakile, Ikenna K. Uchendu, Chidiebere Peter Echieh, Kingsley John Emmanuel, Regina Idu Ejemot-Nwadiaro, Glory Mbe Egom Nja, Adaeze Oreh, Mercy Ogechi Uchenwa, Emmanuel Chukwuma Ufornwa, Ndidi Patience Nwaiwu, Christopher Ogar Ogar, Ani Nkang, Obinna Justice Kabiri, F. Javier Povedano-Montero

**Affiliations:** 1 Medical Laboratory Science Department, Arthur Jarvis University, Akpabuyo, Nigeria; 2 Department of Human Physiology, Arthur Jarvis University, Akpabuyo, Nigeria; 3 Biochemistry Department, Arthur Jarvis University, Akpabuyo, Nigeria; 4 Department of Biological Sciences, Arthur Jarvis University, Akpabuyo, Nigeria; 5 Department of Human Anatomy, Arthur Jarvis University, Akpabuyo, Nigeria; 6 Department of Nursing Science, Arthur Jarvis University, Akpabuyo, Nigeria; 7 Department of Public Health, Arthur Jarvis University, Akpabuyo, Nigeria; 8 Microbiology Department, University of Calabar, Calabar, Nigeria; 9 Department of Optometry, Arthur Jarvis University, Akpabuyo, Nigeria; 10 Department of Medical Laboratory Sciences, University of Nigeria, Enugu Campus, Enugu State, Nigeria; 11 Division of Cardiothoracic Surgery, University of Calabar Teaching Hospital, Calabar, Nigeria; 12 Department of Public Health, Faculty of Allied Medical Sciences, University of Calabar, Calabar, Nigeria; 13 Department of Planning, Research and Statistics, National Blood Service Commission, Abuja, Nigeria; 14 Medical Research Department, Federal Medical Centre, Owerri, Imo State Nigeria; 15 Medical Laboratory Science Department, Imo State University, Owerri, Nigeria; 16 Medical Laboratory Science, University of Calabar, Calabar, Nigeria; 17 Department of Medical Laboratory Science, Imo State University, Owerri, Imo State Nigeria; 18 School of Biomedical and Health Sciences, Universidad Europea de Madrid, Madrid, Spain; 19 Department of Optics V (Optometry and Vision, Faculty of Optics and Optometry, Complutense University of Madrid, Madrid, Spain; 20 Neurology Unit, Hospital 12 de Octubre Research Institute (I+12), Madrid, Spain

**Keywords:** HIV, AIDS, HIV in Nigeria, AIDS in Nigeria, Acquired Immunodeficiency Syndrome, Human immunodeficiency virus

## Abstract

**Background:**

Acquired immunodeficiency syndrome (AIDS) is an acquired defect of the cellular immunity associated with the infection by the human immunodeficiency virus (HIV). The disease has reached pandemic proportion and has been considered a public health concern. This study is aimed at analyzing the trend of HIV/AIDS research in Nigeria.

**Method:**

We used the PUBMED database to a conduct bibliometric analysis of HIV/AIDS-related research in Nigeria from 1986 to 2021 employing “HIV”, “AIDS”, “acquired immunodeficiency syndrome”, “Human immunodeficiency virus”, and “Nigeria” as search description. The most common bibliometric indicators were applied for the selected publications.

**Result:**

The number of scientific research articles retrieved for HIV/AIDS-related research in Nigeria was 2796. Original research was the predominant article type. Articles authored by 4 authors consisted majority of the papers. The University of Ibadan was found to be the most productive institution. Institutions in the United States dominated external production with the University of Maryland at the top. The most utilized journal was PLoS ONE. While Iliyasu Z. was the most productive principal author, Crowel TA. was the overall most productive author with the highest collaborative strength. The keyword analysis using overlay visualization showed a gradual shift from disease characteristics to diagnosis, treatment and prevention. Trend in HIV/AIDS research in Nigeria is increasing yet evolving. Four articles were retracted while two had an expression of concern.

**Conclusion:**

The growth of scientific literature in HIV/AIDS-related research in Nigeria was found to be high and increasing. However, the hotspot analysis still shows more unexplored grey areas in future.

**Supplementary Information:**

The online version contains supplementary material available at 10.1186/s12981-022-00489-6.

## Background

Acquired Immunodeficiency Syndrome (AIDS) is an acquired defect of the cellular immunity associated with infection by the human immunodeficiency virus (HIV), a CD4 positive lymphocyte count of less than 200 cells/micrometer and increased susceptibility to opportunistic infection [1].

The first cases of AIDS were reported in May 1981 in the United States of America by Dr. Michael Gottlieb of the Medical School of Los Angeles, United States, and was followed by an official report by the Centre for Disease Control (CDC) on June 5, 1981. The first victims were five homosexual men who were suffering from unusual pneumonia called Pneumocystis Carinii pneumonia and Kaposi’s sarcoma. The causative organisms were first isolated and named Human T-Lymphotropic Virus type III (HTLV-III) in the US and Lymphadenopathy Associated Virus (LAV) in France [2–4]. Specifically, Luc Montagnier and colleagues in Pasteur Institute France in 1983 first isolated the causative organism [5]. The following year (1984) Robert Gallo of the National Institute of Health isolated the causative organism (HTLV-III) [6, 7]. At the same time, Jay Levy and colleagues at UCSF also independently isolated the virus [8, 9]. However, Robert Gallo was the first to lay a causative link between the virus and AIDS. In May 1986, the international community on taxonomy of viruses chaired by Harold Varmus harmonized and recommended the renaming of the virus with different names to human immunodeficiency virus, following the evidence that they (HTLV-III and LAV) were genetically indistinguishable [10].

On the African Continent, HIV/AIDS was first reported in Uganda, East Africa in 1982 [11].

The first case of HIV and AIDS in Nigeria was identified in 1985 and reported at an international conference in 1986. The first two cases as reported by the Federal Ministry of Health were; a sexually active 13 year-old girl and a female commercial sex worker from a neighboring West African country [2, 4].

**Fig. 1:**
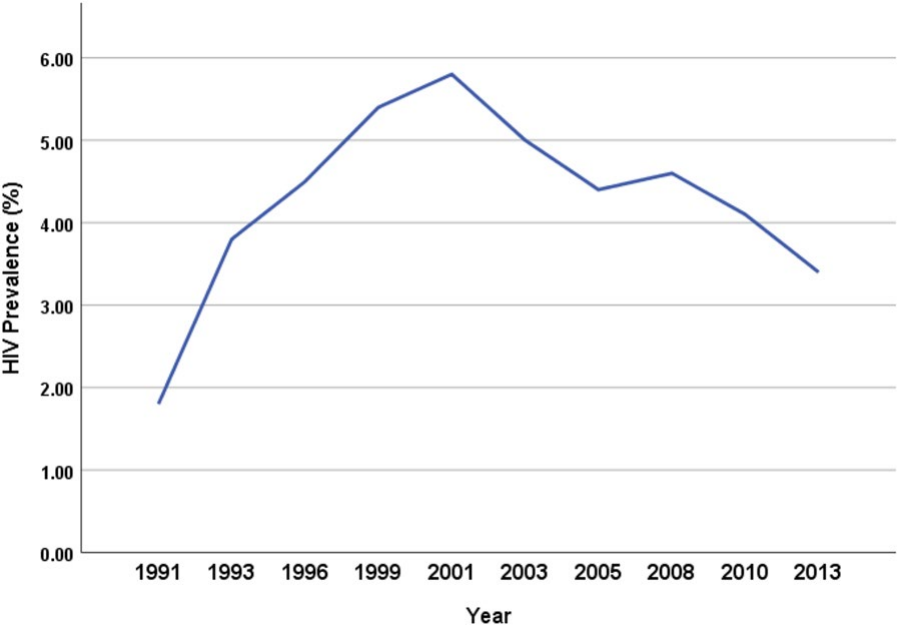
Trend of HIV prevalence over the years

**Fig. 2:**
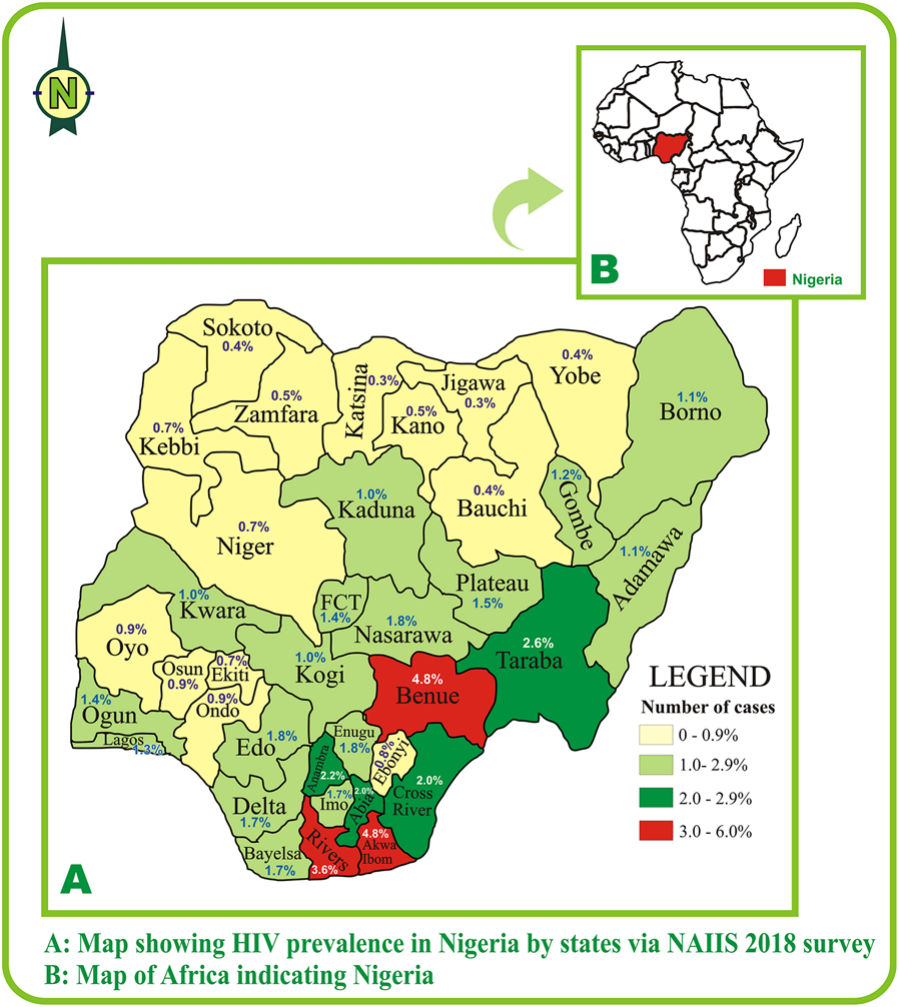
HIV prevalence in Nigeria by states

Nigeria is the most populous African country and the seventh most populous in the world with an estimated population of approximately 206,139.589 people [12, 13]. It is located within the eastern strip of West Africa with an area of 923,768 Km^2^ [14]. Nigeria is a multi-ethnic and culturally diverse federation of 36 autonomous states and the Federal Capital Territory [15]. The first HIV/AIDS sentinel survey was conducted in 1991 with a prevalence of 1.8% which since then increased to 3.8% in 1993, 4.5% in 1996, 5.4% in 1999, and peaked at 5.8% in 2001. Post 2001, decline trend was observed in 2003 (5.0%), 2005 (4.4%), 2008 (4.6%), 2010 (4.1%), 2013 (3.4%) [16, 17] (Fig. 1). Despite the declining prevalence/low prevalence, HIV/AIDS in Nigeria remains a public health concern. Nigeria ranks 4^th^ in global HIV burden with approximately 1.8 million (estimated) persons living with HIV as of 2019 [18–20]. The current national prevalence of HIV in Nigeria is 1.4% and stratification based on states showed the highest prevalence in Akwa Ibom (5.6%), Benue (4.9%), Rivers (3.8%), Taraba (2.7%) and Anambra (2.7%) and the least prevalence in Jigawa (0.3%) and Katsina (0.3%) [21] (Fig. 2).

Bibliometric studies are relevant tools in the social and scientific evaluation of a given discipline within a specified time frame. They serve as proxy markers for the activities in a given field of research. They evaluate progress/growth and identify gaps in research [22, 23]. The performance analysis of a selected study discipline is often done via bibliometrics and social network analysis (SNA). While the bibliometric data computes the basic outputs, the social network analysis interprets the influence of social links and interactions [24].

This study was aimed at identifying the trend as well as the contribution of Nigeria to HIV/AIDS research. The findings of this study is expected to evaluate progress and identify gaps in HIV research in Nigeria as well as give direction to areas of research and research funding.

## Methods

### Data source

The PUBMED database was used for the bibliometric analysis. PUBMED comprises more than 34 million citations for biomedical literature from MEDLINE, life science journals and online books [25]. Ancillary data were retrieved from Google scholar. Retraction watch database was searched to complement PUBMED on retracted articles and those with an expressions of concern [26].

### Data collection

We analyzed the bibliometric data on HIV/AIDS study in the PUBMED published from January 1, 1986 to December 31, 2021. The study period was chosen on the assumption that all research on HIV/AIDS in Nigeria were published from 1986 when the disease was first reported in Nigeria. The search was performed on May 15, 2022. We made use of advanced search in PUBMED using “MESH” terms “HIV” and “AIDS” and applied the following keywords: “HIV” [Title/Abstract] OR “AIDS” [Title/Abstract] OR “Acquired Immunodeficiency Syndrome” [Title/Abstract] OR “Human Immunodeficiency Virus” [Title/Abstract] AND “Nigeria” [Title/Abstract]. We retrieved all data under the above predefined search query without restriction on article type. The retrieved data were used to compute bibliometric indicators. Since PUBMED does not store citation records, we retrieved the citation information about authors and articles via Google scholar. We also re-searched PUBMED using the above search descriptors in addition to “Retraction” and Expression of concern”. We also searched the Retraction database setting the location to Nigeria. Extra detail on search query is presented in the Additional file 1: Table S1.

### Screening protocol and criteria

Only articles with focus on HIV/AIDS in Nigeria were included. Articles that were not focused on HIV/AIDS but mentioned same on passing were excluded as well as those not in Nigeria. There was no restriction on the type of article. Duplicate articles were also removed. Two review groups among the authors independently performed the article selection. Differences in opinion were settled via consensus of both grouping. The full detail of exclusion diagram is presented in the Additional file 2: Fig. S2.

### Visualization of social network analysis

We used the VOSviewer (Center for Science and Technology Studies, Leiden University, The Netherlands) version 1.6.18 to map HIV/AIDS terms and collaboration in the retrieved data from PUBMED.

### Bibliometric indicators

#### Impact factor

The impact factor (IF) is utilized as a measure of the journal’s influence and was originally developed by the Institute for Science Information (Philadelphia PA, USA) as a bibliometric indicator. It is updated annually in the Journal Citation Report (JCR) of Clarivate Analytics and the value is often a marker of prestige. We used JCR data of 2021.

#### Author/institution participation index

WE evaluated the overall 1986–2021 scientific publication in the discipline of HIV/AIDS in Nigeria. It is the number of documents on the topic in question (in this case HIV/AIDS in Nigeria) by an author/institution with respect to the total publications in that domain.

#### Keyword analysis

WE used keyword analysis to ratify the trend of discussion and research in view of the disease characteristics, pathology and treatment.

#### Co-authorship analysis

CO-authorship refers to the interaction of authors contributing to the particular field of study. The co-authorship of papers between authors shows collaboration [24, 27]. The co-authorship network map as generated by VOSviewer show collaborative social network of research fields.

### Bibliometric mapping

Bibliometric mapping was divided into two parts: co-authorship mapping and co-occurrence mapping. Co-authorship refers to the interactions of authors in institutions contributing to the field of study, while co-occurrence refers to relationship among keywords.

The following keys of interpretation are utilized in the visualization of co-authorship network analysis: The size of the nodes or bubbles (circles) within the network corresponds to the frequency or number of documents from an author or institution. Secondly, the lines or arcs between nodes correlate/reflect the existence and intensity of the co-authorship link. Finally, the last legend is the color of the node: VOSviewer clustering algorithm assigns the colors to the nodes based on the estimation of a measure of similarity between them. Consequently, it is safe to conclude that nodes of same color are related. Also, the shorter the distance between two (2) nodes, the closer the relationship between them [24].

## Results

### Results of publication output

We retrieved 2838 publications and only included 2796 publications after removing 9 duplicate publications and 33 publications that were either not related to Nigeria (as in, mentioned Nigeria in passing) or not related to HIV/AIDS (Only mentioned in abstract background) (total of 42). Of these, 92.13% (n = 2576) were original articles, 2.79% (n = 78) were narrative reviews, while 1.14% (n = 32) were systematic reviews. Other forms of publications recorded were Case reports/Case series (0.96%), Perspectives (0.86%), Correspondence/Letters to Editor/Comments on articles (0.71%), Gazettes/Law reviews and other official publications (0.39%), Commentary (0.29%), Erratum/Corrections (0.25%), Conference /Workshop papers (0.18%), Editorials (0.14%), Books/Book chapters (0.11%) and Expressions of concern (0.03%) (Table 1).

**Table 1:** Description of research output within study period based on article type

Article type	Frequencies (%)
Original article	2576 (92.13)
Narrative review	78 (2.79)
Systematic review	32 (1.14)
Case report/ case series	27 (0.96)
Perspective	24 (0.86)
Correspondence/Letter to Editor/Comments on article	20 (0.71)
Gazettes/Law reviews/Other official publications	11 (0.39)
Commentary	8 (0.29)
Erratum/ Corrections	7 (0.25)
Conference/workshop papers	5 (0.18)
Editorial	4 (0.14)
Books/ Book chapters	3 (0.11)
Expression of concern	1 (0.03)
Total	2796

**Fig. 3:**
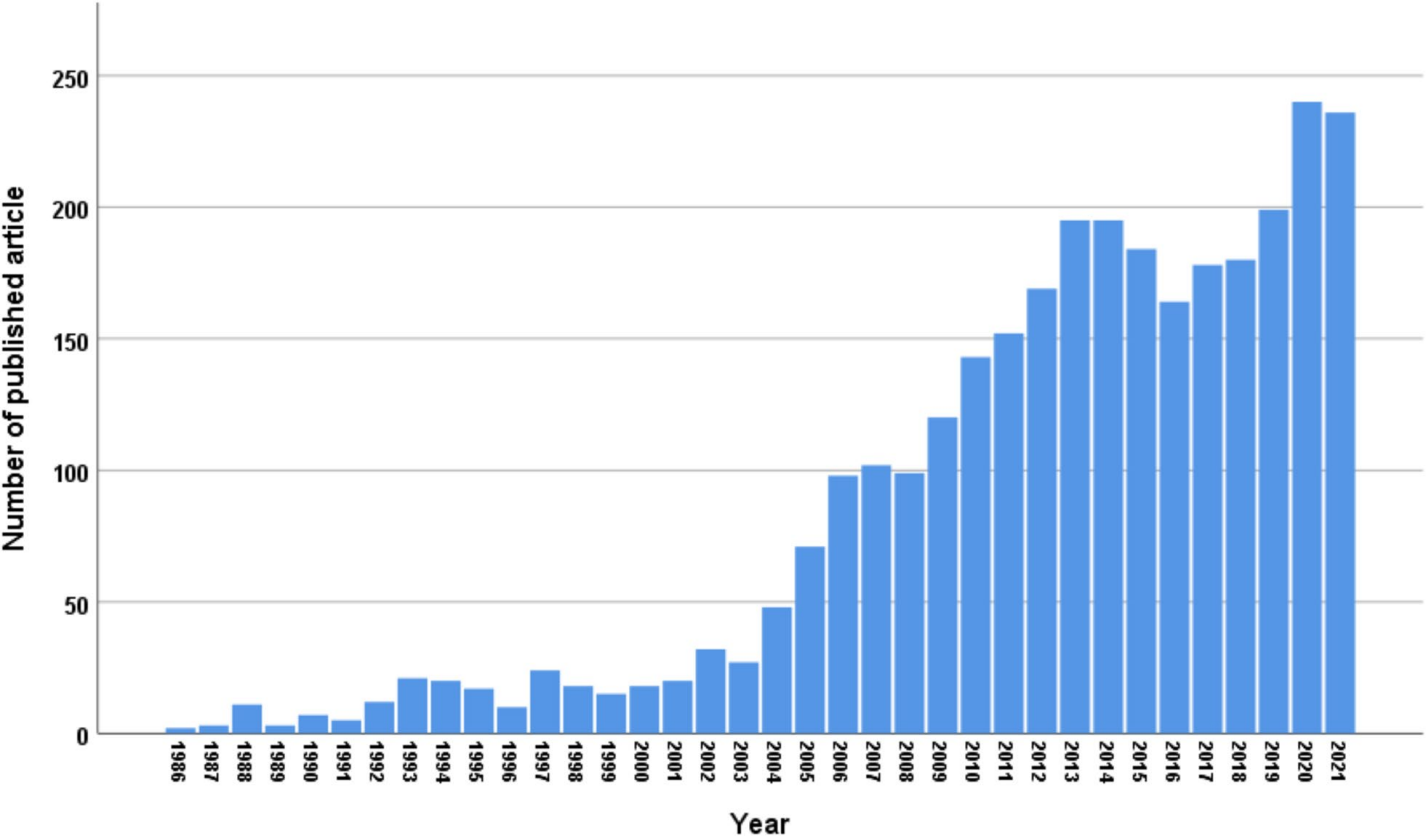
Trend line of publication of HIV/AIDS related literature in Nigeria from 1986 to 2021

The first publications (2 in number) were published in 1986. There was a slow pace of publication of HIV/AIDS related literature from then till the year 2004 when publications shot up more than 20-fold. The tempo of research since then has been sustained and has remained ≥ 150 publications per year after 2011 (Fig. 3).

### Analysis of proportion of articles by number of authors

Our result showed a large span of number of authors per document ranging from single author documents to > 10 authors per document. Furthermore, the result showed that more than half (59.66%; n = 1668) of the published articles were by collaboration of < 6 authors (Table 2). The document with the most authors had 324 author signatures and the most frequent number of signatures was 4.

**Table 2:** Proportion of articles published by respective number of authors

No. of authors	No. of articles (%)
1 author	185 (6.62)
2 authors	339 (12.12)
3 authors	453 (16.2)
4 authors	391 (13.98)
5 authors	300 (10.73)
6 authors	279 (9.98)
7 authors	154 (5.51)
8 authors	155 (5.54)
9 authors	113 (4.04)
10 authors	98 (3.50)
≥ 10 authors	324 (11.59)
Gazettes and other organizational publications^a^	5 (0.18)

^a^Without specific authors

### Analysis of most productive institutions

The top most productive institutions in HIV/AIDS research in Nigeria were represented in Table 3. University of Ibadan, Nigeria was the most productive institution (n = 176), followed by the University of Lagos (n = 112), University of Nigeria Enugu (n = 97), Obafemi Awolowo University (n = 76) and others. Only the first top 8 institutions accounted for more than 25% (25.64%) of the total produced literature. Worthy of note is that University of Ibadan and its affiliated teaching hospital (University College Hospital) made the top list of the most productive institutions. Similarly, University of Nigeria Enugu and its affiliated institution (University of Nigeria Teaching Hospital, Enugu) also made it to the top list. University of Maryland School of Medicine was the only foreign institution that made the top 20 list (n = 47; 1.68%). Out of the top 20 institutions, 10 are federal public universities, 6 are federal tertiary health institutions, 2 federal research institutes, 1 State university resident in Nigeria and a USA-based public land—Grant University (Table 3).

**Table 3:** Top 20 most productive institutions and 5 most productive countries in HIV/AIDS research in Nigeria

Participating institutions	Type of institution	No. of documents (%)
University of Ibadan, Ibadan, Nigeria	Federal University	176 (6.29)
University of Lagos, Lagos, Nigeria	Federal University	112 (4.00)
University of Nigeria, Enugu State, Nigeria	Federal University	97 (3.47)
Obafemi Awolowo University, Ile-Ife, Osun state, Nigeria	Federal University	76 (2.72)
University of Benin Teaching Hospital, Benin City, Edo State, Nigeria	Federal Tertiary Hospital	67 (2.40)
University College Hospital, Ibadan, Oyo State, Nigeria	Federal Tertiary Hospital	67 (2.40)
Jos University Teaching Hospital, Jos, Plateau State, Nigeria	Federal Tertiary Hospital	64 (2.29)
University of Nigeria Teaching Hospital, Enugu, Nigeria	Federal Tertiary Hospital	58 (2.07)
University of Jos, Jos, Plateau State Nigeria	Federal University	57 (2.04)
Institute of Human Virology, Abuja, Nigeria	Federal research Institute	53 (1.89)
University of Maryland, Baltimore, USA	Public land-grant University	47 (1.68)
University of Benin, Benin City, Nigeria	Federal University	43 (1.54)
Nigerian Institute of Medical Research, Yaba, Lagos, Nigeria	Federal Research Institute	37 (1.32)
Bayero University Kano Nigeria	Federal University	34 (1.22)
Nnamdi Azikiwe University Teaching Hospital, Nnewi, Anambra State, Nigeria	Federal Tertiary Hospital	31 (1.11)
Aminu Kano Teaching Hospital Kano, Nigeria	Federal Tertiary Hospital	31 (1.11)
Lagos State University, Lagos, Nigeria	State University	30 (1.07)
University Of Calabar, Calabar, Cross River State Nigeria	Federal University	28 (1.00)
University of Port Harcourt, Port Harcourt, Nigeria	Federal University	28 (1.00)
University of Ilorin, Ilorin, Kwara State, Nigeria	Federal University	27 (0.96)
External participation/collaboration by country		
United States of America		359 (12.84)
South Africa		53 (1.89)
United Kingdom		51 (1.82)
Canada		21 (0.75)
The Netherlands		14 (0.50)

On account of external participating countries, institutions in the USA produced 12.84% (n = 359) of the total published literature. This was followed by South Africa, United Kingdom, Canada and Netherlands (Table 3). Individual analysis of the USA-based institutions showed that the University of Maryland produced 1.6% (n = 45) of the literature followed by US Centre for Disease Control (0.7%; n = 20), Harvard School of Public Health (0.50%; n = 14), Emory University (0.50%; n = 14), Vanderbilt Institute for Global Health (0.46%; n = 13) and Johns Hopkins Bloomberg School of Public Health (0.39%; n = 11). Among the South African based institutions, the top productive was from University of Kwazulu—Natal (0.46%; n = 13) and University of Western Cape Town (0.23%; n = 7). The top participating institution from UK was London School of Hygiene and Tropical Medicine (0.14%; n = 4), while that of Canada and Netherlands were University of Ottawa (0.18%; n = 5) and Maastrich University, respectively (Additional file 3: Table S3).

### Analysis of co-authorship of participating institutions

**Fig. 4:**
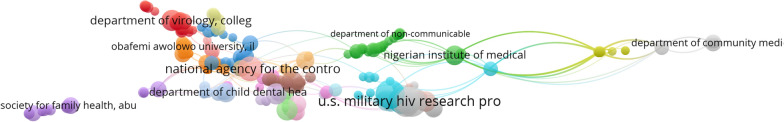
collaborative network among institutions publishing HIV/AIDS related research in Nigeria

Figure 4 shows the collaborative network among institutions publishing HIV/AIDS related research in Nigeria. The threshold for the mapping was set at minimum of 2 collaborations. Of the 645 qualifying institutions, only 367 (56.90%) were connected (had collaboration). The most collaborating institutions with the total link strengths are: US Military HIV Research Program (109 link strength), HJF Medical Research International Abuja (91 LS), Henry M. Jackson Foundation for advancement of Military Medicine USA (85 LS), Institute of Human Virology Abuja (58 LS) and Makerere University Walter Reeds Project Uganda (56 LS).

Among the strongest links of the US Military Research Program Include: Institute of Human Virology University of Maryland, HJF Medical Research International Abuja, Medicine University, Population Council of Nigeria Abuja, National Hospital Abuja, US Army Medical Research, Henry M. Jackson Foundation for the advancement of Military Medicine and Institute of Human Virology Abuja.

However, the overall strongest collaboration (9 link strength) was found between the US Military HIV Research Program and Institute of Human Virology University of Maryland.

### Analysis of sources with highest publication

Table 4 shows the sources with the highest number of HIV/AIDS related research in Nigeria. PLoS ONE, Pan African Medical Journal, African Journal of Reproductive Health, AIDS Care, Nigeria Journal of Medicine, Journal of Acquire Immune Deficiency Syndrome, African Journal of Medicine and Medical Science, Nigeria Journal of Medical Practice, West African Journal of Medicine and African Health Science, consisted the top most productive sources. Among these, 5 of the sources had impact factor (JCR 2021). Two among them (PLoS ONE and Journal of Acquired Immune Deficiency Syndrome) had impact factor greater than 3. Five of the journals are affiliated to Nigeria. All the journals were multidisciplinary medical journals except African Journal of Reproductive health dedicated to reproductive health and AIDS Care and Journal of Acquired Immune Deficiency Syndrome both dedicated to HIV/AIDS research.

**Table 4:** Analysis of sources with the highest number of publications in HIV/AIDS research in Nigeria

Article source	No of documents	Productivity index	Impact factor^a^	Country of origin	Abbreviated journal title
*PLoS ONE*	109	3.90	3.240	USA	PLoS One
*Pan African Medical Journal*	88	3.15	N/A	Kenya/Cameroon	Pan Afr. Med. J
*African Journal of Reproductive Health*	83	2.97	N/A	Nigeria	Afr. J. Reprod Health
*AIDS Care*	81	2.89	2.320	United Kingdom	AIDS Care
*Nigeria Journal of Medicine*	80	2.86	N/A	Nigeria	Niger. J. Med
*Journal of Acquired Immune Deficiency Syndrome*	80	2.86	3.475	USA	J. Acquir. Immune Defic. Syndr
*African Journal of Medicine and Medical Science*	79	2.82	N/A	Nigeria	Afri. J. Med. Med. Sci
*Nigeria Journal of Clinical Practice*	67	2.39	0.968	Nigeria	Niger. J. Clin. Pract
*West African Journal of Medicine*	66	2.36	N/A	Nigeria	West Afr. J. Med
*African Health Science*	63	2.25	0.927	Uganda	Afr. Health Sci

^a^2021 JCR (Clarivate Analytics, 2021)

### Analysis of most cited articles

Table 5 shows the top 10 most cited articles on HIV/AIDS related research in Nigeria. The most cited article was an article on the discriminating attitude and practice of health care workers towards patients published in PLoS Medicine while the second most cited was a randomized control trial on the use of a vaginal gel for the prevention of HIV infection published in PLoS ONE. The rest were research articles on the effectiveness of intervention methods, knowledge and attitude towards HIV infection, quality of life among HIV-infected persons and provision of outreach services. Two among the most cited articles were published in PLoS Medicine, while another 2 were published in PLoS ONE. All the articles were original research. Despite being the 9th and 8th most cited articles, the articles by Abdullahi et al*.* in PLOS Medicine and Swartz et al. in Lancet HIV had the highest number of citation per year; 152 citations per year and 26.5 citations per year, respectively. Next were the 2nd and 1st most cited publications in PLoS ONE and PLoS Medicine with 24 citations per year and 22 citations per year, respectively.

**Table 5:** Top 10 most cited articles in HIV/AIDS research in Nigeria

Article	Authors	Source	Citation	Article type
Discriminatory attitudes and practices by health workers toward patients with HIV/AIDS in Nigeria	Reis C, Heisler M, Amowitz LL, (…) Anyamele C, Iacopino V	PLoS Med. 2005	359	Original article
SAVVY vaginal gel (C31G) for prevention of HIV infection: a randomized controlled trial in Nigeria	Feldblum PJ, Adeiga A, Bakare R, Wevill S, (…), Rountree W	PLoS One. 2008	316	Original article
A school-based AIDS education programme for secondary school students in Nigeria: a review of effectiveness	Fawole IO, Asuzu MC, Oduntan SO, Brieger WR	Health Educ Res. 1999	241	Original article
Sexual networking in the Ekiti district of Nigeria	Orubuloye IO, Caldwell JC, Caldwell P	Stud Fam Plann. 1991	220	Original article
Knowledge, attitudes, beliefs and motivations towards blood donations among blood donors in Lagos, Nigeria	Olaiya MA, Alakija W, Ajala A, Olatunji RO	Transfus Med. 2004	199	Original article
Effectiveness of cellulose sulfate vaginal gel for the prevention of HIV infection: results of a Phase III trial in Nigeria	Halpern V, Ogunsola F, Obunge O, Wang CH, (…) Crucitti T, Abdellati S	PLoS One. 2008	166	Original article
Relationship between depression and quality of life in persons with HIV infection in Nigeria	Adewuya AO, Afolabi MO, Ola BA, (…), Oladipo BF, Fakande I	Int J Psychiatry Med. 2008	161	Original article
The immediate effect of the Same-Sex Marriage Prohibition Act on stigma, discrimination, and engagement on HIV prevention and treatment services in men who have sex with men in Nigeria: analysis of prospective data from the TRUST cohort	Schwartz SR, Nowak RG, Orazulike I, Keshinro B, Ake J, Kennedy S, Njoku O, Blattner WA, Charurat ME, Baral SD; TRUST Study Group	Lancet HIV. 2015	159	Original article
Providing TB and HIV outreach services to internally displaced populations in Northeast Nigeria: Results of a controlled intervention study	Abdullahi SA, Smelyanskaya M, John S, Adamu HI, Ubochioma E, Kennedy I, (…) Stevens R, Creswell J	PLoS Med. 2020	152	Original article
Assessing effects of a media campaign on HIV/AIDS awareness and prevention in Nigeria: results from the VISION Project	Keating J, Meekers D, Adewuyi A	BMC Public Health. 2006	147	Original article

### Analysis of most productive authors by principal author analysis

Table 6 shows the most productive authors in HIVS/AIDS-related publication in Nigeria by principal author analysis. Iliyasu Z, Folayan MO, Ogoina D, Uneke CJ, Olowookere SA, Aliyu MH, Ogunbayo A, Olakunle BO, Daniel OJ, Aliyu G and Agaba PA were the top productive first authors. Thirteen of the 15 authors are affiliated to Nigeria while the other 2 are affiliated to USA.

**Table 6:** Fifteen most productive first authors in HIV/AIDS research in Nigeria (first author analysis)

Author	Country	h-index	Affiliation	No of documents	% of Documents
Iliyasu Z	Nigeria	32	Bayero University, Kano	21	0.75
Folayan M.O	Nigeria	45	Obafemi Awolowo University, Ile Ife	16	0.57
Ogoina D	Nigeria	13	Niger Delta University, Bayelsa	13	0.46
Uneke C.J	Nigeria	NA	Ebonyi State University, Ebonyi	11	0.39
Olowookere S.A	Nigeria	19	Obafemi Awolowo University, Ile Ife	10	0.36
Aliyu M.H	USA	46	Vanderbilt Universty Medical Center, USA	9	0.32
Ogunbayo A	USA	14	Havard University	8	0.29
Olakunde B.O	Nigeria	11	National Agency for the Control of AIDS	8	0.29
Olley B. O.	Nigeria	27	University of Ibadan	8	0.29
Sam-Agudu M.A	Nigeria	21	Institute of Human Virology	8	0.29
Lawson L	Nigeria	24	Bingham University, Nasarawa	8	0.29
Ezeanolue E.E	Nigeria	24	University of Nigeria, Nsukka	8	0.29
Daniel O.J	Nigeria	19	Olabisi Onabanjo University, Ogun	8	0.29
Aliyu G	Nigeria	NA	National Agency for the Control of AIDS	8	0.29
Agaba P.A	Nigeria	24	University of Jos	8	0.29

*NA* not available

### Overall co-authorship analysis of authors

**Fig. 5:**
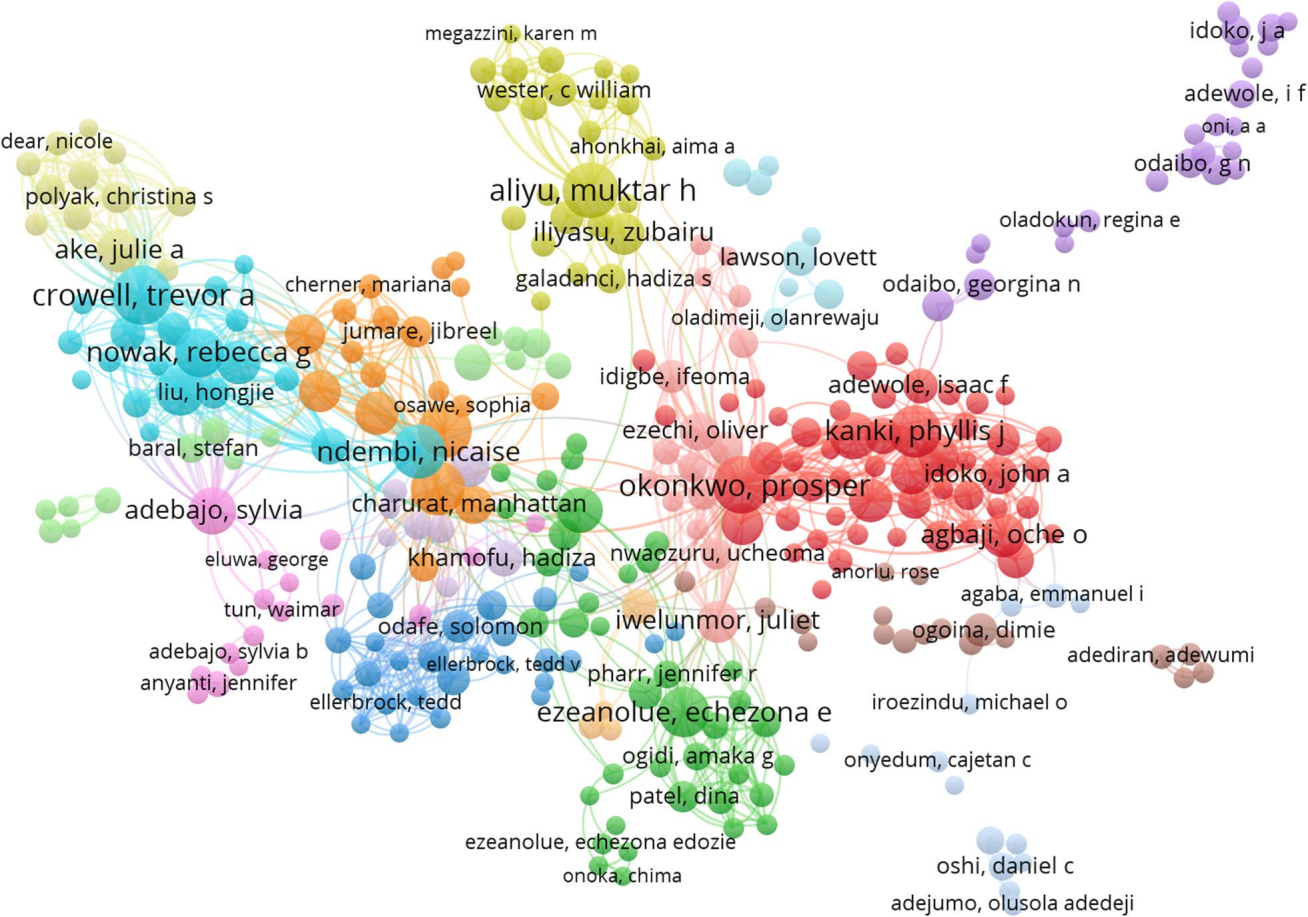
Co-authorship network among authors publishing HIV/AIDS related articles in Nigeria

Figure 5 shows the network of co-authors made up of authors who have published at least five (5) HIV/AIDS-related research in Nigeria. The network contained 316 nodes, 2522 co-authorship links, 7258 total link strength and 16 clusters.

The node symbol represents an author while the node size represents activity/publications of the author, while links between the authors represent relationship between them. Exactly 78 (19.80%) of the 394 authors who met the minimum selection criteria (at least 5 publications) had no connection (collaboration).

Based on total link strength, Crowel TA (361; turquoise cluster), Okonkwo P (284; red cluster), Ndembi N (245; turquoise cluster), Nowak R (241; turquoise cluster), Baral SD (219; turquoise cluster), Dakum P (203; orange cluster), Kanki P (190; red cluster), Charurat MP (179; orange cluster), Aliyu MH (171; golden lemon cluster), Adebajo S (170; lavender cluster) and Ezeanolue E (165; green cluster) were the most influential authors in HIV/AIDS research in Nigeria network. Considering the total number of co-authored articles (both as principal author and as co-authors), Crowell TA (n = 45), Okonkwo P (n = 43), Aliyu MH (n = 40), Ndembi N (n = 38), Dakum P (n = 37), Kanki PJ (n = 34), Ezeanolue E (n = 32), Nowak R (n = 31), Baral SD (n = 30) Adebajo S (n = 29) and Ake JA (n = 24) are in this order the most productive authors. Crowell TA, Okonkwo P and Ndembi N retained the position of the most co-authorship as well as the top total link strength. The three are affiliated to Uniformed Service University USA, Bingham University Nigeria and Africa CDC, respectively (Table 7).

**Table 7:** Analysis of most influential authors by co-authorship collaboration

Author	No of documents	Total link strength	Affiliation	h index
Crowel, Trevor A	45	361	Uniformed Service University, USA	22
Okonkwo, Prosper	43	284	Bingham University, Nigeria	20
Ndemb, Nicaise	38	245	Africa Center for Disease Control	34
Nowak, Rebecca G	31	241	University of Maryland, USA	NA
Baral, Stefan D	30	219	Johns Hopkins School of Public Health	65
Ake, Julie A	24	214	US Military Research Programme	25
Dakum, Patrick	37	203	Institute of Human Virology, Abuja, Nigeria + University of Maryland, USA	NA
Kanki, Phyllis J	34	190	Havard University	NA
Charurat, Manhattan E	25	179	University of Maryland, USA	42
Aliyu, Muktar H	40	171	Vanderbilt University, USA	46
Adebajo, Silvia	29	170	HIV and AIDS Programme, Population Council, Abuja, Nigeria	26
Ezeanolue, Echezona	32	165	University of Nigeria	25

Notably, Aliyu MH (of Vanderbilt University USA) retained the 6th most productive author position by principal author analysis (n = 19) as well as the 3rd most co-authored author (n = 40) while having the 10th highest total link strength. Similarly, Ezeanolue E made it on both list as the 12th most published principal author as well as the 7th most co-authored author and the 12th highest total link strength (Tables 6 and 7).

### Keywords/hotspot analysis

**Fig. 6:**
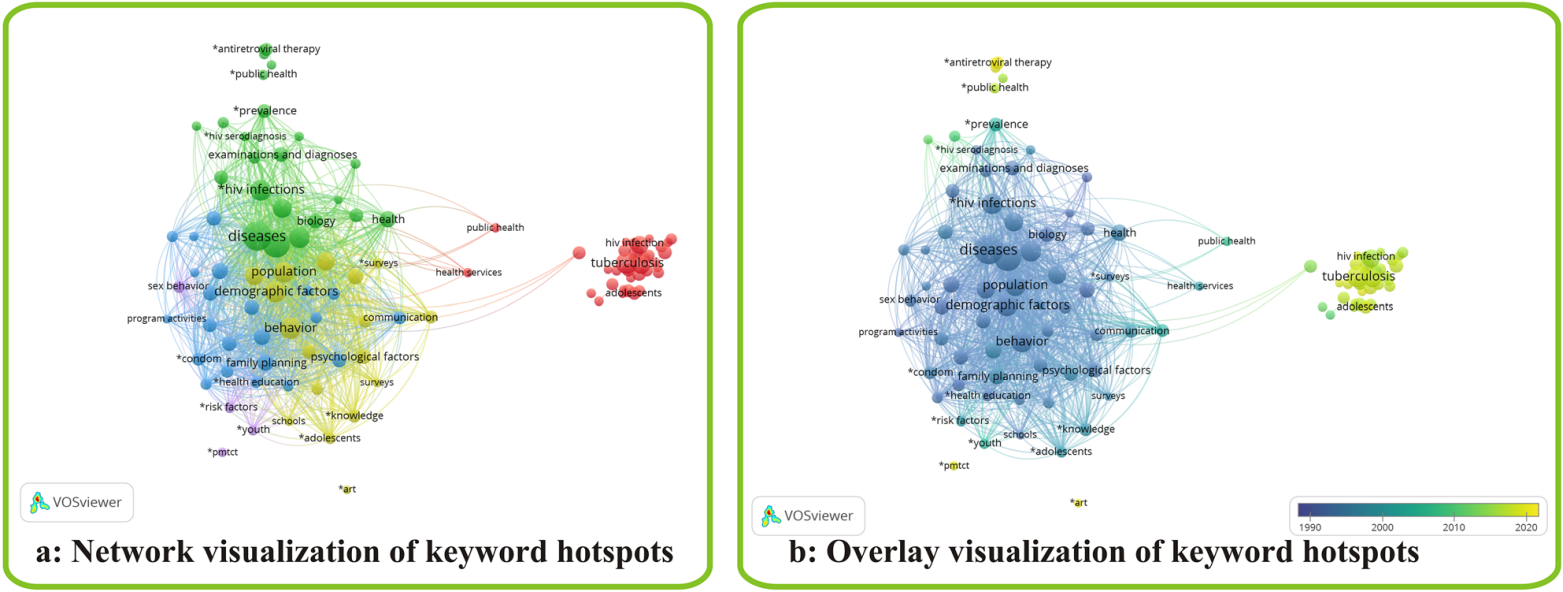
Hotspot analysis of author keywords used in HIV/AIDS related studies in Nigeria

Figure 6 shows hotspot analysis of author keywords used in HIV/AIDS related studies in Nigeria. Keywords appearing more than 10 times were included in the map. Exactly 120 keywords qualified for this. The network visualization stratified the keywords into 5 clusters. Cluster 1 (red) represented treatment, diagnosis, mortality, epidemiology and co-mobility. Tuberculosis (56) and prevalence (41) were the most occurring keywords in cluster 1. However, mortality had higher link strength (70) with other keywords, despite lower occurrence (21). Cluster 2 (green) focused on treatment, epidemiology and co-mobility. Diseases (104) and viral diseases were the most prominent keywords in cluster 2. Cluster 3 (blue) represented keywords associated with the modes of HIV prevention. Education (34), family planning (33), condoms (20) and barrier methods (16) were the most prominent keywords in cluster 3. Cluster 4 (yellow) represented keywords on disease characteristics and demographics. Behavior (55), and demographic factors were the most prominent keywords in cluster 4. However, behavior had the highest link strength (with other keywords) in cluster 4. Cluster 5 (purple) represented keywords associated with risk factors/mode of transmission of HIV. Sex behavior (19) and risk factors (13) were the most occurring keywords in cluster 5. Overall, keywords relating to co-mobility with tuberculosis and HIV prevalence were the most occurring keywords (Fig. 6a).

On the ground of different average appearing year of keywords, VOSviewer under overlay visualization marked keywords included in the map with different colors (Fig. 6b). Keywords in blue appeared earlier than those in green and yellow. Keywords in cluster 1 and a few in cluster 5 appeared in more recent years, revealing epidemiology, antiretroviral therapy and prevention of mother-to-child transmission (PMTCT) as current topics of discussion in HIV/AIDS research in Nigeria (2018.36-2019.60) (Fig. 6b).

Keywords such as antiretroviral therapy and PMTCT showed no links are therefore research areas still open for new researches.

### Analysis of retracted articles and those with expression of concerns

Overall, our analysis found 4 retracted articles and 2 articles with expression of concern in HIV/AIDS related publications in Nigeria. Two of the retracted articles were systematic reviews published in Cochrane Database of Systematic Reviews published by Wiley Publishing Company. The other 2 were a conference paper published in Sexually Transmitted Infections (published by BMJ Publishing) and an original article published in African Journal of AIDS Research (published by Taylor & Francis). The 2 articles with expressions of concern were published in Kidney International (published by Elsevier). Most of the reasons for retractions/expressions of concerns were raised by the authors (Table 8).

**Table 8:** Number of articles retracted or with expression of concern within the study period

Article type	Type of action	Reason for action	Article topic	Date published	Date of action	Citations	Journal name	Publisher
Conference abstract paper	Retraction	At the request of the authors	Seroprevalence of Human immunodeficiency virus (HIV) infection among Tuberculosis (TB) patients attending TB/DOTS Centre Nnewi South East Nigeria	July 1, 2013	July 15, 2014	Nil	Sexually Transmitted Infection	BMJ Publishing
Systematic review	Withdrawal (Retraction)	Temporary removal so that authors may add some studies that were missing	Effectiveness and safety of first-line fixed dose TNF + EMC + EFZ for patients with HIV	July 16, 2008	May 16, 2012	2	Cochrane Database of Systematic Reviews	Wiley
Systematic review	Withdrawal (Retraction)	Temporary removal so that authors may add some studies that were apparently missed	Effectiveness and safety of first-line fixed dose tenofovir + emtricitabine + efavirez for patients with HIV (Pub2)	February 15, 2012	May 16, 2012	12	Cochrane Database of Systematic Reviews	Wiley
Research article	Retraction	Ethical violation (consent) + error in data	Higher risk sexual behaviour among HIV patients receiving antiretroviral treatment in Ibadan, Nigeria	May 1, 2008	March 27, 2014	18	African Journal of AIDS Research	Taylor & Francis
Research article	Expression of concern	Concern raised by authors	Apolipoprotein-1 risk variants and associated kidney phenotypes in an adult HIV cohort in Nigeria	July 1, 2021	November 20, 2022	6	Kidney International	Elsevier
Research article	Expression of concern	Concern raised by authors	APOL1-associated kidney disease in northern Nigerians with treated HIV infection	July 1, 2021	November 20, 2022	Nil	Kidney International	Elsevier

## Discussion

This study provides a quantitative description of HIV/AIDS related research in Nigeria from 1986 to 2021 in PUBMED. The most utilized document type by the authors was original article implying that the subject matter was mostly experimental or clinical.

The trend of research output on HIV/AIDS in Nigeria showed a progressive increase and reassuring trend. However, we found that there was a sluggish growth of HIV/AIDS related literature in Nigeria until 2004 when dramatic growth was observed with an inflection point at about 2008. The earlier lag in scientific productivity could be related to the initial response to the epidemic. Balogun and colleagues [2] identified three major phases in the development of HIV/AIDS epidemic in Nigeria. First, there was an era of absolute official and personal denial of the presence of HIV/AIDS in Nigeria (1981–1986) [2]. A publication in 1987 [28] reported that government officials insisted that AIDS was non-existent in Nigeria even after 18 other African countries had reported the disease. Secondly, there was an era of skepticism and indifference (1986–1997) which was overwhelmed with misconceptions. People described AIDS literally as “American Idea to Destroy Sex” and some even bragged that Africans were immune to it. Finally, the last phase was the era of reality (1997 till date).

The core journals that served as sources for HIV/AIDS related publication in Nigeria were PLoS ONE, Pan African Medical Journal, African Journal of Reproductive Health, AIDS Care, Journal of Acquired Immune Deficiency Syndrome, African Journal of Medicine and Medical Science. These journals could possibly be avenues for future breakthroughs in HIV/AIDS research in Nigeria. More so, the patronage of PLoS ONE with a high impact factor may not be unconnected to the editorial policy of the journal. PLoS ONE emphasizes scientific rigor of a research work over novelty unlike other journals within that category [29].

The article “Discriminating attitude and practice by health workers towards patients with HIV/AIDS in Nigeria” published in PLoS Medicine was the most cited article. The article was dedicated to assessing the attitude of health care workers toward HIV infected persons in Nigeria; refusal to attend to HIV/AIDS patients, suitability to attend to HIV/AIDS patients in general ward and the need to disclose HIV status to all health workers. The second most cited article was on phases 3 double-blind randomized clinical trial of a vaginal gel intended for prevention of HIV infection. The article with the most citations per year was an article published in Lancet HIV that dwelt on effect of same sex marriage. All the top 10 most cited articles were published in journals with impact factor > 3. The feat of the article published in Lancet is not surprising considering the high impact factor (16.070) and wider coverage of the journal. However, there have been counter argument on the translation of journal impact factor to individual article citation [30].

University of Ibadan, University of Lagos and University of Nigeria, Enugu and Obafemi Awolowo University were the most outstanding in terms of productivity in HIV/AIDS related research in Nigeria. The above institutions have been consistently documented to occupy the top five (5) positions in researches in biotechnology research [31], Lassa fever research [32] and overall research [33]. They are among the early Federal Universities in Nigeria. The University of Ibadan is the first university in Nigeria founded as University College Ibadan (part of University of London) in 1948 and was later converted to indigenous university in 1962 [34]. It has been ranked 1st in Nigeria and 1172nd in the world (2022–2023 World University Ranking) [35]. University of Lagos is a public federal university founded in 1962, and is ranked 3rd in Nigeria and 1924th globally [36]. University of Nigeria, Enugu was formally opened in 1960 as the first indigenous university, and is ranked 2nd in Nigeria and 1775th globally [37]. The top 6 institutions are all institutions located in southern Nigeria.

Institutions in the United States dominated external publications in HIV/AIDS research in Nigeria and accounted for 12.84% of all publications. Prominent among these institutions were University of Maryland USA and Centre for Disease Control. The United States has been in the forefront of HIV/AIDS research/treatment and funding in Nigeria. This has been via national and corporate funding. For instance, the US President’s Emergency Plan for AIDS Relief (PEPFAR) has shown the highest commitment in HIV/AIDS research, diagnosis and antiretroviral therapies [38, 39]. The AIDS Prevention Initiative (APIN) funded by Bill and Melinda Gates Foundation has offered substantial funds in the form of grants for HIV research and treatment [40]. The dominance of USA in various fields of study is well documented [22, 23]. The United States has been reported to have committed 3.45% of her GDP to research and development (R & D) [41].

Crowel TA, Okonkwo P, Aliyu MH, Ndembi N, DakumP,Kanki P, Ezeanolue E, Nowak RG, Baral SD, Adebajo S, Charurat ME and Ake JA were the all-round most productive authors in HIV/AIDS related research in Nigeria. Collaborative link analysis presented Crowel TA, Okonkwo P, Ndenbi N, Nowak RG, Baral BD, Ake JA, Dakum P, Kanki PJ, and others as the most influential in terms of diversity of links. Prominent to note is Crowel TA who is the most productive author as well as the one with the highest collaborative strength. On the other hand, analysis of authors’ contribution based on principal author (first author) analysis showed Iliyasu Z, Folayan MO, Ogoina D, Uneke CJ, Olowookere SA, Aliyu MH, Ogunbayo A, Olakunde BO, Olley BO, Sam-Agudu MA, Lawson L, Ezeanolue EE, Daniel OJ, Aliyu G and Agaba PA to be the most productive authors. The above authors in the two categories are core to HIV research in Nigeria and are likely to have tremendous impact in HIV/AIDS research in future.

Analysis of the co-authorship collaboration network showed that Aliyu MH and Ezeanolue were the only authors in the top list of principal authors who had high link strength of collaboration. Also, most of the top authors with high collaborative strength were affiliated to institutions in the United States and some Nigerian government agencies with external funding. Only Okonkwo P of Bingham University (Private University) and Ezeanolue E (University of Nigeria) were the only top list authors from Universities in Nigeria in terms of collaboration. This observation is further corroborated by the institutional collaboration network analysis. The major collaborating institutions were USA-based institutions and externally funded federal agencies. Most of the federal universities only had inter-university collaboration and were in periphery of the network, hence, their exclusion in the network link map. There is poor funding of research in Nigeria, especially with regards to Nigerian Universities. Nigeria spends only 0.13% of her GDP on research and development (R&D) [41]. This is far below the recommended average of 2.3% by Organization for Economic Co-operation and Development (OECD). The only major source of academic funding in Nigerian public universities is TETFUND (Tertiary Education Trust Fund) which is limited and often rationed funds based on grant applications with limited scope (and don’t even cover private universities). The bulk of research in tertiary institutions in Nigeria are self-funded by academic staff, graduate students, staff-in-training and are driven by the demand for publication towards career development [42–45].

The keyword analysis using overlay visualization showed a gradual shift from disease characteristics to diagnosis, treatment and prevention. The current discussions are on mapping current epidemiology, administration of antiretroviral therapy and the prevention of mother-to-child-transmission of HIV. For instance, there have been varying current discussions on trends, predictors, spatial patterns, knowledge and the reduction of mother-to-child transmission of HIV in Nigeria [46–50]. Often, the first response to an epidemic is to characterize the disease followed by diagnosis and possible means of amelioration/cure. With no absolute curative means to HIV and poor access to ameliorative means, preventive measures have becomes the ultimate means to combat the disease especially in resource limited setting such as Nigeria.

The analysis of articles that had post-publication remarks showed 4 articles withdrawn (retracted) and 2 with an expression of concern. We observed that some of the articles continued to accumulate citations even after they were retracted. For example, the article “Higher risk sexual behavior among HIV patients receiving antiretroviral treatment in Ibadan Nigeria” had 18 citations in total, 7 of which occurred after retraction on May 16, 2014. This observation supports the argument and submission of some researchers [51] that most authors do not read most of the articles they cite. Rather, they copy from an already cited page. Simkin and Roychowdhury [52] have even put a number to it by concluding in their research article “Read before you cite” that only approximately 20% of citers read the original article.

The present study may contain some limitations which are inherent in bibliometric studies. First, the criteria mapped out by the PUBMED database themselves determine the subsequent product of the studied materials. Secondly, local journals that were not indexed in PUBMED within the study period would have been missed. We might have excluded HIV/AIDS research articles in Nigeria if the authors did not include our specific search descriptors. Lastly, we were limited to use PUBMED a free to use database, we may have missed some articles indexed only elsewhere. However, we believe the output is a true representation of research trend in the study domain.

## Conclusion

Irrespective of the inherent limitations, we believe that this study has made available a significant representation of the trends in HIV/AIDS research in Nigeria. We have shown that research on HIV/AIDS in Nigeria had a slow start, possibly due to delay in accepting the reality of the disease, but has grown significantly over time. As current treatment approaches are yet to be curative, it highlights the fact that there remains enormous research potential for the future. The major collaborations were found to be from oversea institutions majorly the United States of America.

## Supplementary Information

**Additional file 1: Table S1**. Search Strategy For The Study In Pubmed.

**Additional file 2: Fig. S2.**. Screening protocol of retrieved data

**Additional file 3:** **Table S3**. Countries with the most published articles.

## Data Availability

Datasets generated and analyzed in this study are within the article. The primary source of data, PUBMED is publicly available.

## Abbreviations

AIDS: Acquired immunodeficiency syndrome
HIV: Human immunodeficiency virus
CDC: Center for Disease Control
HTLV-III: Human T-Lymphotropic virus Type III
LAV: Lymphadenopathy associated virus
SNA: Social Network Analysis
JCR: Journal Citation Report
PEPFAR: President’s Emergency Plan for AIDS relief
APIN: AIDS Prevention in Nigeria
R & D: Research and development
TETFUND: Tertiary Education Trust Fund
OECD: Organization of Economic Co-operation and development
