# Supplementary material for: Thirty-five years (1986–2021) of HIV/AIDS in Nigeria: bibliometric and scoping analysis
Source: AIDS Res Ther. 2022 Dec 21;19:64. doi: 10.1186/s12981-022-00489-6 (PMC9768871; doi:10.1186/s12981-022-00489-6)
Supplement: Supplementary file 1 — Additional file 1: Table S1. Search Strategy For The Study In Pubmed. [file 12981_2022_489_MOESM1_ESM.docx]

| MESH TERMS | “HIV” |
| --- | --- |
|  | “AIDS” |
| SEARCH KEYWORDS | “HIV” OR “AIDS” OR “Acquired Immunodeficiency Syndrome” OR “Human Immunodeficiency Virus” AND “Nigeria” |
| Document type | No limitation |
| Field | Title/Abstract |
| Time limit | January 1, 1986 to December 31, 2021 |
| Language Limitation | None |

**Table S1: SEARCH STRATEGY FOR THE STUDY IN PUBMED**
