# Supplementary figures and images for: Thirty-five years (1986–2021) of HIV/AIDS in Nigeria: bibliometric and scoping analysis
Source: AIDS Res Ther. 2022 Dec 21;19:64. doi: 10.1186/s12981-022-00489-6 (PMC9768871; doi:10.1186/s12981-022-00489-6)

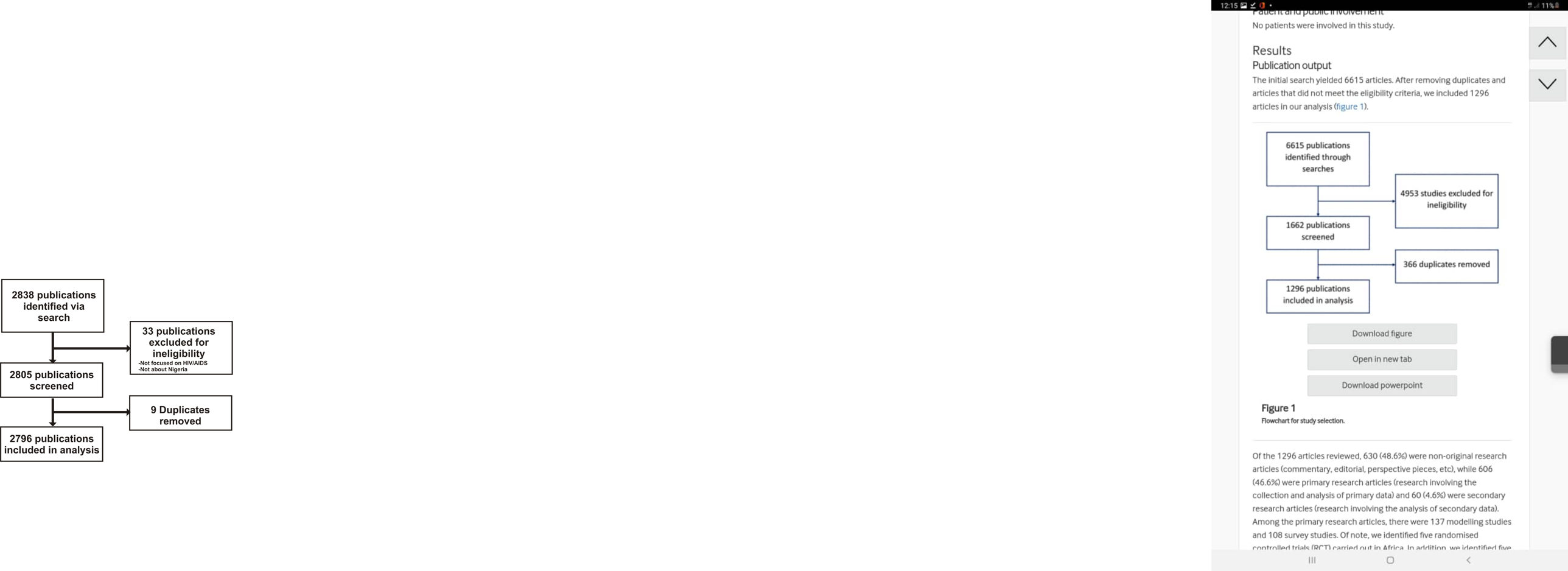

Supplement: Supplementary file 2 — Additional file 2: Fig. S2.. Screening protocol of retrieved data [file 12981_2022_489_MOESM2_ESM.png]
