# Supplementary material for: Thirty-five years (1986–2021) of HIV/AIDS in Nigeria: bibliometric and scoping analysis
Source: AIDS Res Ther. 2022 Dec 21;19:64. doi: 10.1186/s12981-022-00489-6 (PMC9768871; doi:10.1186/s12981-022-00489-6)
Supplement: Supplementary file 3 — Additional file 3: Table S3. Countries with the most published articles. [file 12981_2022_489_MOESM3_ESM.docx]

**Table S3: COUNTRIES WITH THE MOST PUBLISHED ARTICLES**

| **COUNTRIES** | **NO. OF PUBLICATIONS** |
| --- | --- |
| UNITED STATE OF AMERICA | 359 |
| SOUTH AFRICA | 53 |
| UNITED KINGDOM | 51 |
| CANADA | 21 |
| NETHERLANDS | 14 |
| **STRATIFICATION OF SOME TOP INSTITUTIONS FOR EACH TOP COUNTRY** |  |
| **UNITED STATE OF AMERICA** |  |
| University of Maryland School of Medicine, Baltimore, USA | 45 |
| Centers for Disease Control and Prevention, USA | 20 |
| Harvard School of Public Health, Boston, MA, USA. | 14 |
| Emory University, Atlanta, GA, USA. | 14 |
| Vanderbilt Institute for Global Health, Nashville, TN, USA | 13 |
| John Hopkins Bloomberg School of Public Health, Baltimore, MD 21205, USA. | 11 |
|  |  |
| **SOUTH AFRICA** |  |
| University of KwaZulu-Natal, South Africa. | 13 |
| University of the Western Cape, Cape Town, South Africa | 7 |
| University of the Witwatersrand, Johannesburg, South Africa. | 6 |
| University of Cape Town, Cape Town, South Africa. | 6 |
| University of Stellenbosch, Stellenbosch, South Africa | 4 |
|  |  |
| **UNITED KINGDOM** |  |
| London School of Hygiene and Tropical Medicine, UK | 4 |
| Imperial College, London, Norfolk Place W2 1PG, UK | 3 |
| University of East Anglia, Norwich, UK | 3 |
| Institute for Health Research, University of Bedfordshire, UK. | 3 |
| University of Fort, Hare East, London, UK | 2 |
| University of North South Wales, UK | 2 |
|  |  |
| **CANADA** |  |
| University of Ottawa, Ottawa, Ontario, Canada | 5 |
| University of Windsor, Canada | 3 |
| University of Saskatchewan-Saskatoon, Canada | 2 |
| University of Ontario, Canada | 2 |
| University of Toronto. Canada. | 1 |
| University of Manitoba, Winnipeg, Canada | 1 |
|  |  |
| **NETHERLANDS** |  |
| Maastricht University, Maastricht, The Netherlands | 3 |
| University of Amsterdam, Netherlands | 3 |
| Radboud University Medical Center, Nijmegen, The Netherlands. | 2 |
| Utrecht University, Utrecht, Netherlands | 2 |
| Vrije University, Amsterdam, Netherland | 1 |
| Amsterdam institute of Global Health, Netherland | 1 |
